# Quantitative Determination of Marker Compounds and Fingerprint Analysis of the Seeds of *Vernonia anthelmintica*

**DOI:** 10.1155/2020/8859425

**Published:** 2020-10-30

**Authors:** Zulipiya Maimaiti, Ablajan Turak, Qing Ling Ma, Geyu Liu, Haji Akbar Aisa

**Affiliations:** ^1^Key Laboratory of Plant Resources and Chemistry of Arid Zone, Xinjiang Technical Institute of Physics and Chemistry, Chinese Academy of Sciences, Urumqi 830011, China; ^2^University of the Chinese Academy of Sciences, Beijing 100039, China; ^3^State Key Laboratory Basis of Xinjiang Indigenous Medicinal Plants Resource Utilization, Xinjiang Technical Institute of Physics and Chemistry, Chinese Academy of Sciences, Urumqi 830011, China

## Abstract

In traditional Chinese medicine, the seeds of *Vernonia anthelmintica* (L.) Willd. have been widely used for treatment of cough, skin diseases, diarrhea, fever, schistosomiasis, amoebic dysentery, and gastrointestinal problems, especially in the treatment of vitiligo for thousands of years in China. In this study, an effective, reliable, and accurate high-performance liquid chromatography diode array detector (HPLC-DAD) method was developed for quantitative analysis of 3 marker bioactive compounds and chemical fingerprint of the seeds of *V. anthelmintica*. Data corresponding to common peak areas and HPLC chromatographic fingerprints were analyzed by exploratory hierarchical cluster analysis (HCA) and principal component analysis (PCA) to extract information of the most significant variables contributing to characterization and classification of the analyzed samples. Based on variety and origin, the high-performance thin layer chromatography (HPTLC) method validated the chemical fingerprint results used to screen the in vitro antioxidant activity of *V. anthelmintica*. The results show that the developed method has potential application values for the quality consistency evaluation and identification of similar instant *V. anthelmintica* samples. When considered collectively, this research results provide a scientific basis for the improvement of standardization and specification of *V. anthelmintica* medicinal materials and provide a pathway for the development and utilization of references for the identification of *V. anthelmintica* herbs.

## 1. Introduction

Adequate and accepted research methodology for evaluating traditional Chinese medicine (TCM) has attracted much attention in recent years [1, 2]. However, the chemical composition of TCM is very complex, with multicomponent, multitarget, multichannel integration and other pharmacodynamic effects [3], making the quality control of natural drugs become a worldwide problem. The quality and efficacy of TCM were determined by the types, contents, and proportions of these secondary metabolites [4].

*Vernonia anthelmintica* has been widely used and applied in the fields of medicine, food, and industry [5]. *V. anthelmintica* is an annual herbaceous species of the *Asteraceae* family, which is widely cultivated in high-altitude areas of southern Xinjiang and small regions in Pakistan and India [6]. It has long been used as a valuable traditional medicine in China for the treatment of cough, skin diseases, diarrhea [7], fever [8], schistosomiasis, amoebic dysentery, and gastrointestinal problems [9]. As one of the most important traditional Chinese medicines, it also has analgesic, anti-inflammatory [7], anthelmintic, and antibacterial effects [10]. Especially, the seeds of this plant have a long history in the treatment of vitiligo [11]. These pharmacological activities of *V. anthelmintica* are closely related to its chemical constituents. In recent years, many research studies have reported that *V. anthelmintica* contains many kinds of bioactive components such as sesquiterpenoids, flavonoids [11], triterpenes, steroids [9, 12], and caffeoylquinic acids [6, 13, 14].The effective constituents in *V. anthelmintica* could be affected by some factors such as cultivation area, climate (temperature, humidity, light, and wind), geography, harvest time, and storage. Therefore, it is necessary to clarify the *V. anthelmintica* components and evaluate the quality standard.

The chemical information of plant extracts can be revealed by analytical and chemical techniques such as chromatograms, spectrograms, and other graphs [15, 16]. It can potentially characterize both the marker components and the unknown components in a complex system [17]. Both the US Food and Drug Administration [18] and European Medicines Agency (EMEA) recommended this strategy to assess the quality and consistency of botanical products. The State Food and Drug Administration of China (SFDA) has also stated that all the injections made from herbal medicines should be standardized by chromatographic fingerprinting [19]. Moreover, the SFDA has also suggested that all the herbal chromatograms should be evaluated by their similarities, a commonly employed approach based on calculating the correlative coefficient of original data for botanical products over the past decade [20]. HPLC fingerprinting emerges to be the most widely used one attributed to its convenience and efficiency [21], and it has been widely used for quality control of TCM [22]. According to the best of our knowledge, there are no previous reports on the development of a fingerprint study of *V. anthelmintica* herb profiles to distinguish their geographical origins in various *V. anthelmintica* herb-producing countries. In the previous studies about this plant, we found that three dicaffeoyl quinic acids (CQAs) play an important role in the treatment of vitiligo [23, 24], the 3 CQAs are an isomer and hence there is also the possibility of interchanging each other, and they are high in *V. anthelmintica*. Therefore, the quantitative determination of the 3 CQAs as marker compounds was studied in this article, and chromatographic fingerprinting was established by combination with a basic conventional analytical, highly precise, accurate, and novel pattern recognition method (HPLC-DAD) for quality control of *V. anthelmintica* herbs from different countries of origin.

Thin-layer chromatography (TLC) is a simple, economical, and analytical technique that commonly used to screen low-molecular-weight compounds in complicated pharmaceutical, environmental, and food samples and has taken precedence over other chromatographic approaches such as gas chromatography (GC) and HPLC because of its flexibility, cheapness, accessibility, simplicity, lower consumption of solvents and reagents, and ability to simultaneously handle dozens of samples [25, 26]. As sophisticated instrumentation and high-performance adsorbent layers have been developed for sample analysis and chromatogram and derivatization evaluation, HPTLC and chromatogram development have become fairly popular [27]. As an effective, facile, and rapid method for analyzing complicated mixtures, among the many HPTLC applications, its utilization is of particular interest in fingerprint analysis.

**Figure 1:**
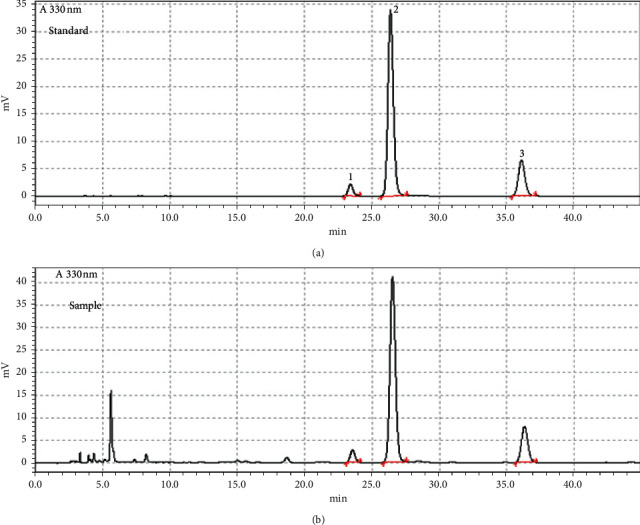
HPLC chromatogram: (a) standard and (b) sample (Peak 1: 3,4-CQA, Peak 2: 3,5-CQA, and Peak 3: 4,5-CQA).

The objective of this study was to establish a simple, sensitive, accurate, and efficient HPLC-DAD analytical fingerprint method of *V. anthelmintica* chromatographic fingerprints using HPLC combined with HPTLC, which was conducted at equal pace for quality control and identification of *V. anthelmintica*. The chemical fingerprint builds a characteristic chemical profile of *V. anthelmintica* or a material that contributes to its identification. The chromatograms of extracted samples from different areas of China and Pakistan were compared visually and analyzed by similarity evaluation. Moreover, three components in 26 batches of *V. anthelmintica* were simultaneously quantified by the HPLC method. The 3 main compounds (Figure 1) are 3,4-O-dicaffeoyl quinic acid (3,4-CQA), 3,5-O-dicaffeoyl quinic acid (3,5-CQA), and 4,5-O-dicaffeoyl quinic acid (4,5-CQA) and were determined simultaneously, and 21 peaks were selected as the common peaks to evaluate the similarities among several *V. anthelmintica* samples collected from different origins in China and Pakistan. The similarity evaluation results showed that location and area differences influenced the quality of the samples. Then, the antioxidant activities of these samples were evaluated by 1,1-diphenyl-2-picrylhydrazyl radical scavenging assay as another parameter to evaluate the quality of *V. anthelmintica*.

## 2. Materials and Methods

### 2.1. Chemicals and Materials

HPLC grade acetonitrile from Merck (Darmstadt, Germany), HPLC grade phosphorous acid from Sigma-Aldrich (Steinheim, Germany), and Wahaha pure water were purchased. Reference compounds for 3,4-CQA (batch number: P0343, purity: >98%) were obtained from Shanghai Youche biotechnology Co., LTD. 3,5-CQA (batch number: 111782–201706, purity: >98%) and 4,5-CQA (batch number: 111894-201102, purity: >98%) were obtained from the Chinese Food and Drug Accreditation Institute. 1,1-Diphenyl-2-picrylhydrazyl (DPPH˙) free radical was obtained from Munich, Germany, and 2,2′-azino-bis(3-ethylbenzothiazoline-6-sulfonic acid) (ABTS∗+) free radical was obtained from Sigma. All other chemicals and solvents used were of analytical grade. Normal-phase silica gel 60 F254 HPTLC glass plates (Merck, Darmstadt, Germany) of a size of 20 × 10 cm were used to perform separation, and Automatic TLC Sampler 4 (ATS4; CAMAG, Muttenz, Switzerland) was used for sampling.

### 2.2. Plant Materials and Preparation of Standard Solutions and Sample Solutions

#### 2.2.1. Plant Materials

A total of 26 batches of seeds of *V. anthelmintica*, which were collected from three major areas—Aksu and Hotan provinces of China and Pakistan (Table 1)—were used for establishing the chemical fingerprinting. The samples were authenticated by Prof. Feng Ying, Associate Researcher, Institute of Ecological Geography of Xinjiang, Chinese Academy of Sciences.

#### 2.2.2. Standard Solution Preparation

Stock standard solutions were prepared by adding accurately weighed standard substances and dissolving with ethanol in water (60 : 40, v/v), containing 0.007264 mg/mL of 3,4-CQA, 0.095212 mg/mL of 3,5-CQA, and 0.022117 mg/mL of 4,5-CQA. Then, the standard solution was diluted to three different concentrations and then filtered through a 0.45 *μ*m membrane prior to injection.

#### 2.2.3. Sample Preparation

The crude samples of *V. anthelmintica* seeds were dried and milled into a powder and sieved through a No. 40 mesh screen. Add precisely the weighed 1.0 g of the dried powder to a 250 mL conical flask. Add accurately 100 mL of 60% ethanol solution, weigh and soak for 30 min, and then heat reflux in an 80°C water bath for 40 min. Weigh again and replenish the lost weight with 60% ethanol solution, shake well, and filtrate through a 0.45 *μ*m membrane filter.

### 2.3. Instrumentation and Chromatographic Conditions

#### 2.3.1. HPLC Conditions for Fingerprinting and Similarity Analysis

The chromatographic analysis was achieved using the Waters e295 Separations Module (Wasters, USA) with a binary pump, an ultraviolet/visible detector, and a column temperature controller. The system control and data analysis were processed with Waters Empower software (Waters, USA). Reversed-phase separation was performed on a Waters HSS T3 (4.6 × 250 mm, 5 *μ*m; Waters, USA) column at 40°C. The mobile phases comprised (A) 0.3% phosphorous acid in water and (B) acetonitrile. The sample was injected (10 *μ*L injection volume) onto the column and eluted at a flow rate of 1 mL/min according to the following gradients: initial 5% B; 0–15 min/5–16% B; 15–40 min/15–23% B; 40–65 min/23–40% B; 65–70 min/40–45% B; 70–75 min/45–80% B; and 75–80 min/80–80% B. Ultraviolet detection was set as follows: 0–28 min/210 nm; 28–28.5 min/230–250 nm; 28.5–29 min/230–250 nm; 29–29.5 min/250–260 nm; 29.5–30 min/260–270 nm; 30–32 min/270 nm; 32–34 min/270–280 nm; 34–40 min/280–230 nm; 40–70 min/230–210 nm; and 70–80 min/210 nm.

#### 2.3.2. HPLC Conditions for Quantitative Determination of the Three Marker Compounds

An Agilent 1260 series HPLC instrument (Agilent, USA) equipped with a DAD detector (Agilent, USA) was used for chromatographic analysis of quantitative determination of the three marker compounds. The system control and data analysis were processed using Agilent OpenLAB CDS ChemStation software (Agilent, USA). The chromatographic separation was performed on a Waters HSS T3 (4.6 × 250 mm, 5 *μ*m, Waters, USA) column at 40°C: (A) 0.3% phosphorous acid in water and (B) acetonitrile as mobile phases. The sample was injected (5 *μ*L injection volume) onto the column and eluted at a flow rate of 1 mL/min according to the following gradients: initial 16% B; 0–40 min/16–18% B; 40–43 min/18–80% B; and 43–48 min/80–80% B. Ultraviolet detection was set to 330 nm.

#### 2.3.3. Determination of Antioxidant Activity

The 5–8 *μ*L of *V. anthelmintica* samples was applied to 20 cm × 10 cm silica gel HPTLC plates as an 18 mm band by utilizing Automatic TLC Sampler 4. An ethyl acetate formic acid-glacial acetic acid-water (30 : 1 1 : 1, v/v/v/v) mixture was used to develop plates in a saturated vertical twin chamber for 30 min, until the bands reached the distance of 70 mm. A hairdryer was used to dry the developed plates for 5–10 minutes, which were immediately dipped into 0.5 % solutions of ABTS∗+ and DPPH˙ in hydrous ethanol using Chromatogram Immersion Device III (CAMAG). UV light was applied to the sample at 366 nm, with white light below and above the plate. The developed plates were photographed before and after they were derivatized with either 0.4% w/v DPPH˙ solution or ABTS∗+ solution. Before photographing, plates were placed in a dark environment for 30 minutes to wait for derivatization of ABTS∗+ and DPPH˙ solution. The reproducibility between the plates and high-quality images was ensured by fixing the parameters captured using the winCATS imaging software (CAMAG, Switzerland). Video Scan Digital Image Evaluation software (CAMAG, Switzerland) was used to perform quantitative analysis of HPTLC and was set to identify fluorescent bands. To process images further, the photos were stored in TIF file format.

### 2.4. Date Analysis

#### 2.4.1. Establishment of HPLC Fingerprint and Similarity Analysis

In the recent years, the advancement of chromatographic and spectral fingerprints plays an important role in the quality control of complex herbal medicines [20]. The chromatographic fingerprint method was highly recommended by the SFDA [20] for evaluating the similarity analysis (SA) of traditional Chinese herb medicine, which accurately calculates the similarity from the correlation coefficient [28, 29] and/or cosine value of the vectorial angle of the original data. SA was thus carried out to determine the degree of similarity or dissimilarity of samples from each other [30]. Therefore, the fingerprint analysis of 26 batches of *V. anthelmintica* was performed using professional software named Similarity Evaluation System for Chromatographic fingerprint of Traditional Chinese Medicine (Version 2004A; National Committee of Pharmacopoeia, China). This reference chromatogram system could reflect the similarity of distribution ratio of the chemical composition accurately. In generally, combination with chromatographic fingerprint analysis methods for quality control has great potential in the application of *V. anthelmintica*.

#### 2.4.2. Hierarchical Clustering Analysis

**Figure 2:**
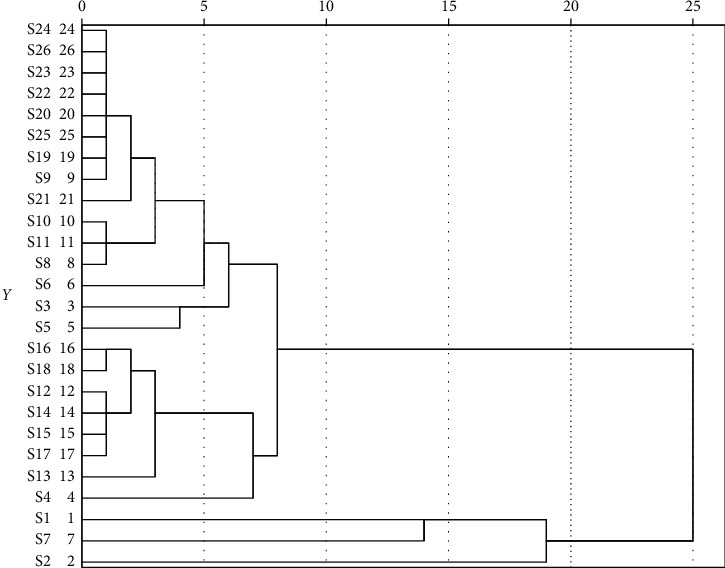
Results of HCA of 26 instant *V. anthelmintica* samples.

By using hierarchical clustering analysis (HCA), the natural clustering of samples can be found according to the fingerprint and all the samples were grouped into different clusters. In this case, HCA was performed on 1–26 samples to analyze the data from HPLC chromatograms carried on the statistics using software SPSS 25.0 (SPSS for Windows 10.0; SPSS Corporation, USA). The clustering analysis models between-groups linkage method as the amalgamation rule and the cosine method as the metric were used to establish clusters. However, no information is provided about clusters or groups of *V. anthelmintica*. The HCA results are shown in Figure 2 as a tree structure diagram providing clearer visualization of data in a high-dimensional matrix.

#### 2.4.3. Principal Component Analysis

In this study, principal components analysis (PCA) and soft independent modelling of class analogy (SIMCA 13) were applied to the similarity matrixes. A 19-row, 26-column data matrix was created and introduced into the software. Scatter plots were defined by the interrelation between each principal component (PCs) for visualization of the data matrix. The purpose of this analysis was to determine the underlying information from multivariate original data by converting and reducing the dimensions of the original data matrix for samples and variables into the product of two matrices, scores (T), and loadings (P), while containing the same information as the original data. The information about the samples is presented in the form of scores, while loadings focus on the variables that have the most influence over the difference between groups of samples.

## 3. Results and Discussion

### 3.1. Validation of the HPLC Method of Quantitative Analysis, Calibration Curves, and Limits of Detection

The quantitative determination of the three marker compounds 3,4-CQA, 3,5-CQA, and 4,5-CQA in 26 batches of *V. anthelmintica* seeds was carried out using a HPLC-DAD, ensuring that the fingerprints are representative and authentic. The use of the optimal linear gradient methods resulted in a good separation of the three analytes and an excellent fingerprint chromatogram with a total analysis time below 40 min for separation of the three marker compounds (Figure 1) under the best detection wavelength conditions with 330 nm. To determine the content of the compounds, first, the linearity of this method was evaluated. Standard solutions were prepared by diluting a specific volume of the standard solutions to get several concentrations. The regression equations of the 3 compounds were calculated in the form of *y* = *ax* + *b*, where *y* and *x* are the peak area and concentration, respectively. The contents of the 3 compounds were calculated by one-point external standard method. The retention times, regression equations, and linear range of the 3 compounds are shown in Table 2. The correlation coefficient of standard curves of the 3 compounds showed they all have good linear correlation in the linear range. The detection limits (LOD) under the present HPLC-DAD method were estimated to be the lowest concentration of the compounds required to produce the signals, which were at least three times stronger than the noise signal (S/N ≥ 3). The LOD and LOQ for the three marker compounds were found to be less than 0.18, 0.14, and 0.15 ng and 0.95, 0.68, and 0.77 ng, which indicated that the analytical method was acceptable with sufficient sensitivity. The HPLC analysis of samples was validated with precision, repeatability, and stability tests. Intraday precision and repeatability as well as intraday stability of the HPLC method were determined and expressed by the relative standard deviation (RSD) values of the average relative retention times (RRT) and relative peak areas (RPA) of the 3 peaks. The intraday precision variation of the RRT and RPA of the 3 peaks was less than 0.20% and 0.30%, respectively. The stability test was evaluated by analysis of the same sample solution at room temperature with different time intervals (0, 2, 4, 6, 8, 10, and 12 h), and the RSD values of RRT and RPA of the 3 peaks were below 0.80% and 0.40%, respectively. It means the sample solution was stable within 12 h. The repeatability test was calculated by analysis of six independently prepared solutions of the same sample. The RSD values of RRT and RPA did not reach 1.00% and 1.20%, respectively. For the recovery test, the standards of three compounds mixture solutions were added according to 1.0 g of powder from seven batches of the same herb samples, and then the standards were extracted, processed, and quantified of three components in the medicinal materials. The RSD values of recovery of the 3 compounds were less than 2.00%. The results of the precision, stability, repeatability, and recovery tests are shown in Table 3.

### 3.2. Method Validation of HPLC Fingerprint Analysis

**Figure 3:**
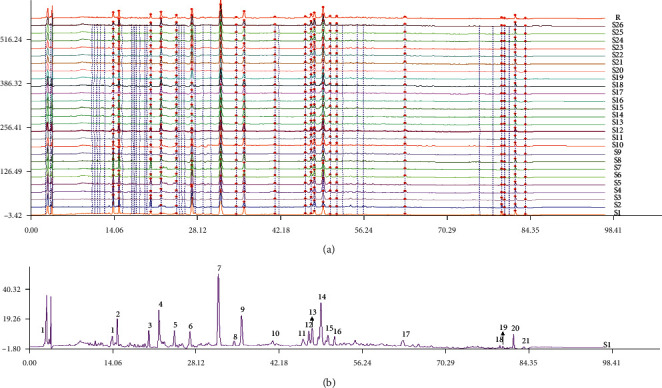
HPLC fingerprint of 26 batches of *V. anthelmintica* samples (a) and the reference fingerprint of *V. anthelmintica*. (b).

HPLC-DAD analysis was used to obtain the chromatographic fingerprints reflecting the complex mixture compound in the samples. Chromatographic fingerprints were generated for 26 samples using the optimized HPLC-DAD method, and the similarities of these 21 peaks were discovered in each separate sample and the contents of CQAs were determined, and the results are shown in Table 4 and Figure 3.

Table 4 shows that samples from Hotan City 2015 and Aksu City in 2015 (S1 and S2) had relatively low similarities (less than 0.90) and the others were over 0.90, implying that the storage time (the storage time is longer than 3 years) influences the quality of samples. According to the results of fingerprint analysis, the total content of the 3 marker compounds in the S1 samples (Total CQA were 1.3403) is the highest, and in the S2 samples, it (Total CQA were 0.8714) is the lowest. This is probably due to the difference in the ecological environment and the preservation time.

### 3.3. Hierarchical Clustering Analysis

Two main analytical procedures, hierarchical cluster analysis (HCA) and principal component analysis (PCA), have been constructed with the use of chemometrics to create the pattern of herbal composition in the form of a mathematical model, and they are widely used for the classification study in the field of food and herbal medicine research [31]. The application of chemometric techniques can greatly improve the quality of the fingerprint obtained from complex chromatographic or spectroscopic profiles.

The HCA procedure could find the natural clusters of samples based on their fingerprints, and all the samples were clearly divided into different clusters. In this case, the linkage between groups was introduced and the cosine measure was calculated. HCA results are shown in Figure 2. Depending on the distance, the 26 batches of samples shown in a dendrogram could be divided into three clusters, which means that the processing procedures caused changes in the composition and/or content of components in *V. anthelmintica*. Samples S1, S2, and S7 were grouped into cluster I. Samples S4, S13, S17, S15, S14, S12, S18, and S16 were grouped into cluster II, and the other samples were grouped into cluster III. In the cluster I herbs, cosine values were above 0.75, and, in the other cluster herbs, were above 0.90. The result suggested the main contents and distribution of the main components were different in different *V. anthelmintica* samples, which would result in their different efficacies. In this study, the correlation coefficient between samples was calculated, and the amalgamation rule and the cosine method were performed to group the samples with low distance as a cluster. A dendrogram was used to monitor the nearest distance between sample clusters.

### 3.4. Principal Component Analysis

To evaluate whether the fingerprint profiles can effectively distinguish 26 batches of *V. anthelmintica* samples, PCA was carried out because it has a good ability to summarize multivariate variation.

**Figure 4:**
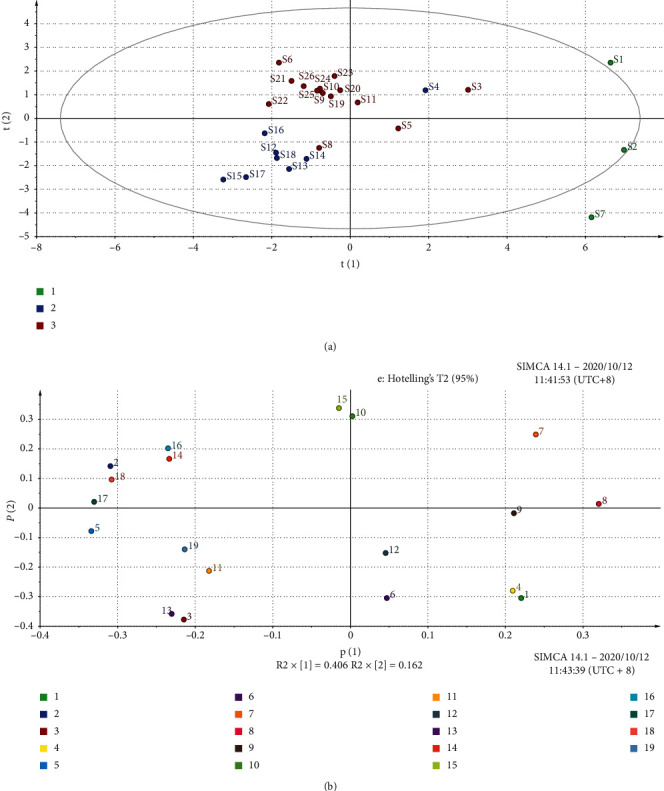
The score plot of the PCA for the 26 samples (a) and the loadings plot of PCA for 26 characteristic peaks (b).

PCA was employed by using the Simca13 software in which the common 21 peaks of the 26 batches of *V. anthelmintica* were chosen as variables to obtain the scores instead of the full spectrum of fingerprints without any preprocessing. The results of scores and loadings scatter plots based on the differences in their HPLC-DAD fingerprints are illustrated in Figure 4. The scatter points showed that the samples could be classified into three groups. Among them, samples in the second group and in the third group were closely clustered, while the samples in the first group were relatively dispersed. The PC1 versus PC2 biplot in Figure 4 accounted for 56.73% data variance (PC1 = 40.55%, PC2 = 16.18%), and two clusters of *V. anthelmintica* were identified. The result of the PCA was in good agreement with SA and HCA, which showed the main contents and distribution of the main component differences to some extent. A variable is placed the farther from the origin, the higher contribution of that variable to the PCA model. According to their loadings, the scatter plot of the scores (Figure 4(b)) indicated that Peak 7 might have more influence on the discrimination of the sample from different sources. PC1 was strongly influenced by the peak 7, 1, 4, and 9 loading with positive values, and PC2 was influenced by peak 7, 15, 16, and 14 loading with positive values. In our study, the SA and PCA gave the same results. Comparing the SA with the PCA, PCA could give a more visual comparison of the chromatograms, and the SA could afford a more quantitative comparison of the samples. Fingerprint analysis assisted by pattern recognition techniques is a potential strategy for the authentication and differentiation of herbal medicines.

### 3.5. High-Performance Thin-Layer Chromatography

**Figure 5:**
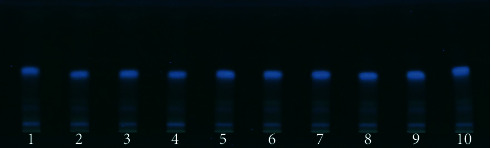
Developed TLC plate of *V. anthelmintica* under UV 366 nm.

The performance of TLC using a sorbent with a homogeneous particle size of 5 *μ*L and with a narrow particle size distribution has been confirmed to be superior to that of the conventional TLC plate, which was used in many pharmacopoeias for species authentication of traditional medicine. Chromatographic and spectral fingerprint analysis has been proved as a very useful and feasible approach, with the advancements of the methods are simple, flexible, fast and less costly, efficient separation techniques for both qualitative and quantitative analysis, enabling simultaneous analysis of many substances in herbal medicines in the minimal time requirement [32]. *V. anthelmintica* contains more complex chemical composition. A large number of samples, such as 10 batches of samples, must be used to accurately determine the characteristic identity of compounds to disclose distinctions among complicated mixtures. Therefore, in this study, the HPTLC plate was used to establish a TLC fingerprinting method. The chromatographic conditions, particularly the developing solvents (i.e., types of solvents, and ratios), were carefully optimized before the 10 batches (S1～S10) of crude drug samples were analyzed.  Figure 5 shows the chromatogram photo documentation of the 10 batches of sample (S1–S10) extracts at 366 nm.

The peak formation of the extracts was observed under UV light, and retention factor (*R*_f_) values of the extracts are given, and the results showed a good separation for all compounds in *V. anthelmintica*.

**Figure 6:**
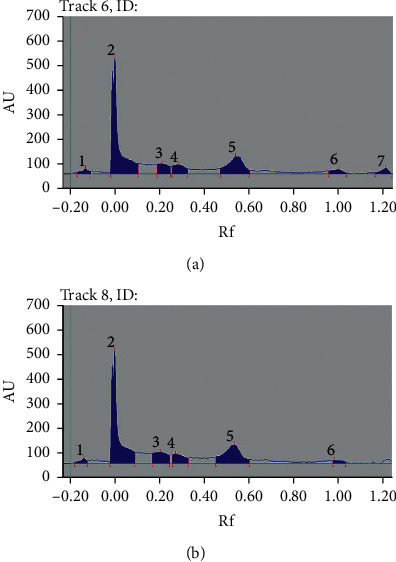
HPTLC fingerprint profile of *V. anthelmintica*: (a) sample 1 and (b) sample 8.

The HPTLC fingerprinting of the 60% ethanol extracts of *V. anthelmintica* revealed 9 peaks in 5 *μ*L volume.  Figure 6 shows the presence of various unknown compounds with *R*_f_ values of −0.15, −0.17, 0.12, 0.21, 0.3, 0.45, 0.55, 1.02, and 1.2, respectively.

### 3.6. In Vitro Antioxidant Activity and Chromatographic Band Visualization

To confirm the active components deduced from fingerprint efficacy relationship analysis, TLC bioautography was performed after separation of antioxidant compounds by thin layer chromatography. Free radical DPPH˙ scavenging activity was observed visually as white yellow zones against the purple background on the plate.  Figure 5 shows a profile of antioxidant fractions of *V. anthelmintica* under visible light. Fractions were observed to have DPPH˙ scavenging activity. The same stained TLC plate was also inspected under UV (366 nm) (Figure 5).

It has been discovered that HPTLC combined with biodetection is particularly helpful in identifying and detecting natural antioxidants. This method first separates the components of natural mixtures on a TLC plate as the adsorbent bed, and subsequently, ABTS∗+ or DPPH˙ jsolution are applied by spraying or dipping the plates into the solution.

In our work, HPTLC combined with a postderivatization DPPH˙ assay and ABTS∗+ assay was successfully used to detect the active potential antioxidative for each phenolic component separated from *V. anthelmintica* in the TLC plate. A direct ABTS∗+ and DPPH˙ assay were used to assess the free radical scavenging activity of *V. anthelmintica*. As a stable free radical with a deep pink colour, DPPH˙ becomes white if the antioxidants present in the sample reduce it. Thus, antioxidant activities of compounds in the sample separated and emerged as white spots and contrast with the pink background above the plate obtained after dipping the HPTLC chromatogram in DPPH˙ solution.

**Figure 7:**
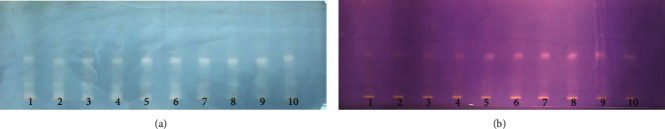
The free radical scavenging activity: (a) after dipping in ABTS∗+ solution and (b) after dipping in DPPH˙ solution; spot volumes 5 *μ*L.

ABTS∗+ is a catalase substrate, and ABTS/ABTS∗+ has a redox potential of 0.68 V, which is prone to electron transfer shift and generates the stable green free radical ABTS∗+ [33]. However, our study indicated that there are much more potent antioxidants in the investigated samples from S1 to S10. Higher amounts of samples contained other antioxidants, the individual compounds in the extract mixtures. This work also clarifies the versatility and flexibility of a normalized HPTLC system as a useful tool in the drug discovery process. The method developed in this work can also be used for the bioassay-guided isolation of unknown natural antioxidants in extract mixtures and the subsequent identification of components utilizing postchromatographic mass spectrometry analysis techniques. The comparison between the colour intensity and area of white bands of crude drugs acquired by phenolic acid-normalized solutions after spraying with an ethanolic DPPH˙ solution (Figure 7) was used to assess the free radical scavenging activity degree within extracts in phenolic acid. In this work, we confirmed that plant extracts positive for free radical scavenging activity were found to be highly correlated with polyphenolic content. The radical scavenging capacity of *V. anthelmintica* might be related mostly to their phenolic hydroxyl groups. The peaks with Rf values between −0.15 and 0.55 of 10 batch samples showed a certain antioxidant activity, which means all 10 batch samples contain polyphenolic compounds. Especially, peaks with Rf values of 0.55 showed strong antioxidant activity. However, according to Figure 7(b), the white colour in samples 1,2,3,4, and 10 are lighter than other 5 samples, which means the content of polyphenolic compounds in these five samples is lower than the others. However, in the HPTLC plate under UV 366 nm, it is hard to find this difference. Thus, the defect of HPTLC fingerprint can be effectively supplemented by in vitro antioxidant activity.

## 4. Conclusions

Chromatographic fingerprint analysis has become an effective and comprehensive evaluation method for quality control of complex TCM and plant extracts, and for species differentiation. In the present study, chromatographic fingerprint analysis and simultaneous determination of three marker dicaffeoyl quinic acids in *V. anthelmintica* were performed using the HPLC-DAD. Nineteen characteristic fingerprint peaks were selected to evaluate the similarities and qualities among 26 batches of *V. anthelmintica* by chemometrics methods including SA, HCA, and PCA. The results clearly demonstrated that the analytical method of HPLC-DAD was reasonable in linearity, repeatability, precision, stability, and recovery; therefore, it could provide valuable quantitative information for the quality assessment of *V. anthelmintica*. The PCA indicated good differentiation of samples with 56.73% of the variation by the first two PCs. With chemometric methods of SA, HCA, and PCA, the 26 samples were objectively grouped into three clusters and the peak 7 played dominating roles. At the same time, we tested the crude drug antioxidant activity using HPTLC-DPPH and HPTLC-ABTS∗+ experiments. Our experimental results regarding antioxidant activity clarified that HPTLC combined with ABTS∗+ and DPPH˙ is a meaningful and powerful tool to comprehensively examine the inhibitory activity and potential antioxidants in traditional Chinese medicine.

## Figures and Tables

**Table 1:** Raw material samples: geographical origin and acquisition time.

Sample	Cultivation area	Time
S1	Hotan	2015
S2	Aksu	2015
S3	Pakistan	2015
S4	Hotan	2016
S5	Hotan	2017
S6	Hotan	2018
S7	Aksu	2018
S8	Pakistan	2018
S9	Hotan A1	2017
S10	Hotan A2	2017
S11	Hotan A3	2017
S12	Hotan A4	2017
S13	Hotan A5	2017
S14	Hotan A6	2017
S15	Hotan A7	2017
S16	Hotan A8	2017
S17	Hotan A9	2017
S18	Hotan A10	2017
S19	Hotan A11	2017
S20	Hotan A12	2017
S21	Hotan A13	2017
S22	Hotan A14	2017
S23	Hotan A15	2017
S24	Hotan A16	2017
S25	Hotan A17	2017
S26	Hotan A18	2017

A1 to A18 denote different bathes of samples.

**Table 2:** Retention time, regression equation, and linear range of three marker compounds.

Compounds	*t*_*R*_ (min)	Regression equation	Linear range (*μ*g/*μ*L)	*R* ^2^
3,4-CQA	23.92	*y* = 2520181.57X − 2996.82	0.011–0.110	0.9999
3,5-CQA	26.92	*y* = 3625124.51X − 29075.73	0.137–1.365	0.9999
4,5-CQA	36.91	*y* = 3624350.26 − 11314.32	0.033–0.325	0.9999

**Table 3:** Analytical results of precision, stability, and reproducibility test of three marker compounds.

Compounds	RSD of RRT (%)			RSD of RPA (%)			Recovery (%, *n* = 7)				
	Precision	Stability	Repeatability	Precision	Stability	Repeatability	Spiked (*μ*g)	Original (*μ*g)	Detected (*μ*g)	Recovery (%)	RSD (%)
3,4-CQA	0.16	0.73	0.94	0.21	0.28	0.96	14.53	16.05	30.52	99.60	1.69
3,5-CQA	0.15	0.69	0.89	0.14	0.32	1.13	190.42	185.42	390.26	107.57	0.76
4,5-CQA	0.14	0.74	0.95	0.22	0.25	0.87	44.34	43.18	88.01	101.10	1.11

**Table 4:** Content and similarity of CQAs in 26 samples of *V. anthelmintica*.

Sample no.	Content of CQA				Similarity
	3,4-CQA	3,5-CQA	4,5-CQA	Total CQA^a^	
S1	0.0378	1.1548	0.1477	1.3403	0.886
S2	0.0569	0.6615	0.1530	0.8714	0.868
S3	0.0587	0.8332	0.1609	1.0528	0.933
S4	0.0348	0.9310	0.1264	1.0921	0.961
S5	0.0423	0.9102	0.2614	1.2138	0.981
S6	0.0388	0.8244	0.1712	1.0343	0.977
S7	0.0559	0.8046	0.1921	1.0526	0.928
S8	0.0493	0.8441	0.2528	1.1462	0.969
S9	0.0399	0.8956	0.1776	1.1130	0.99
S10	0.0400	0.8953	0.1832	1.1186	0.988
S11	0.0405	0.8597	0.2150	1.1152	0.987
S12	0.0427	0.9438	0.1909	1.1774	0.925
S13	0.0402	0.8580	0.2009	1.0992	0.934
S14	0.4161	0.9121	0.2097	1.5379	0.916
S15	0.0412	0.9753	0.1881	1.2046	0.97
S16	0.0372	0.8253	0.1944	1.0570	0.971
S17	0.0403	0.8605	0.2113	1.1122	0.964
S18	0.0367	0.8615	0.1616	1.0598	0.965
S19	0.0348	0.8348	0.1823	1.0519	0.978
S20	0.0368	0.9713	0.1822	1.1903	0.969
S21	0.0355	1.0096	0.1645	1.2096	0.967
S22	0.0356	0.9694	0.1706	1.1756	0.97
S23	0.0379	1.0281	0.1769	1.2430	0.967
S24	0.0368	1.0037	0.1759	1.2163	0.977
S25	0.0382	1.0255	0.1905	1.2543	0.979
S26	0.0383	1.0230	0.1797	1.2410	0.976

a: Total CQA = 3,4-CQA contents + 3,5-CQA contents + 4,5-CQA contents; b: For each constituent, the significant difference of mean content within the same column was less than 0.05 (*p* < 0.05).

## Data Availability

All data generated or analyzed during this study are included within the article.
